# Supplementary figures and images for: IL6 and IL6R as Prognostic Biomarkers in Colorectal Cancer
Source: Biomolecules. 2024 Dec 19;14(12):1629. doi: 10.3390/biom14121629 (PMC11727588; doi:10.3390/biom14121629)

Supplementary Figure 1

A

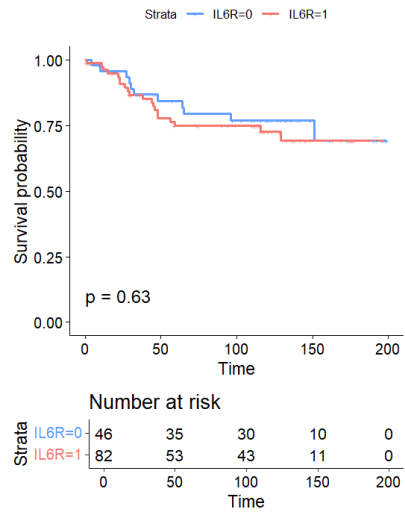

B

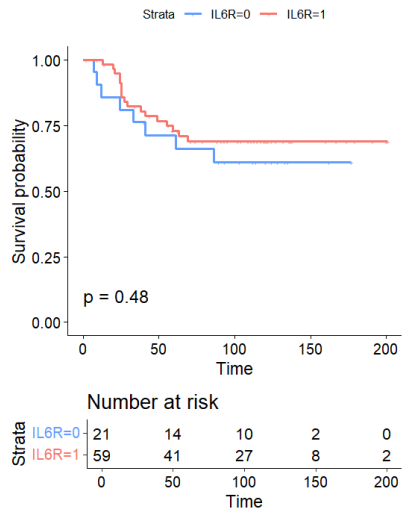

C

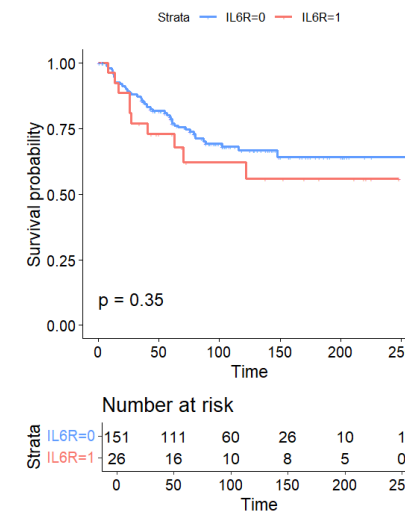

D

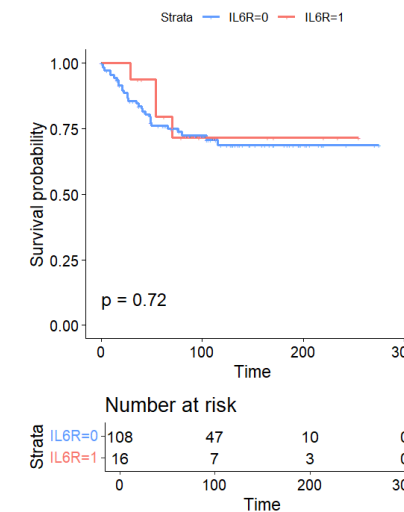

Supplement: Supplementary file 1 [file biomolecules-14-01629-s001.zip › Biomolecules_Supplementary Figure 1.pdf]

Supplementary Figure 4

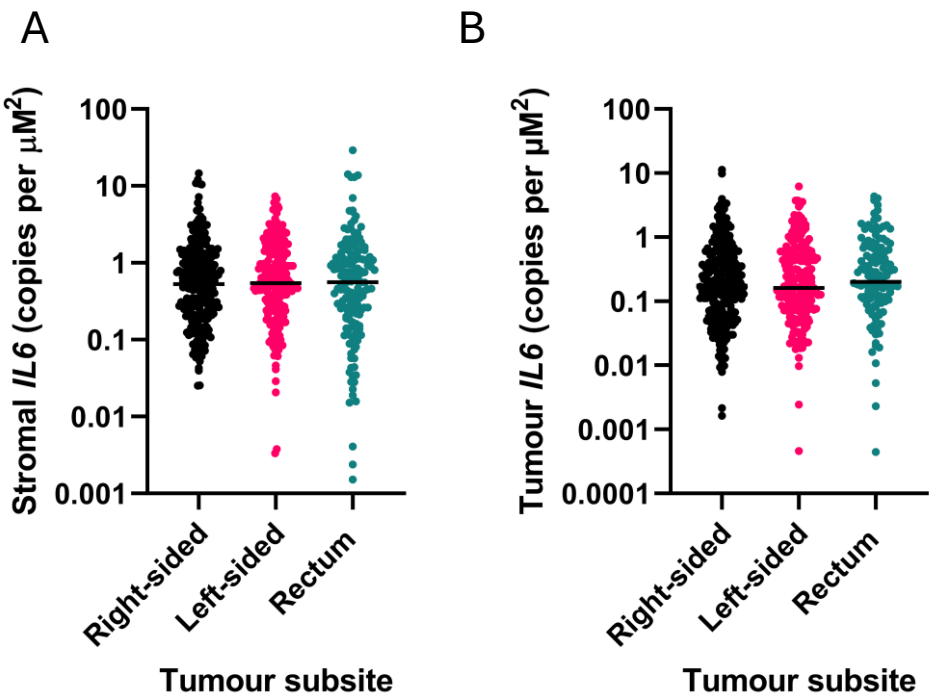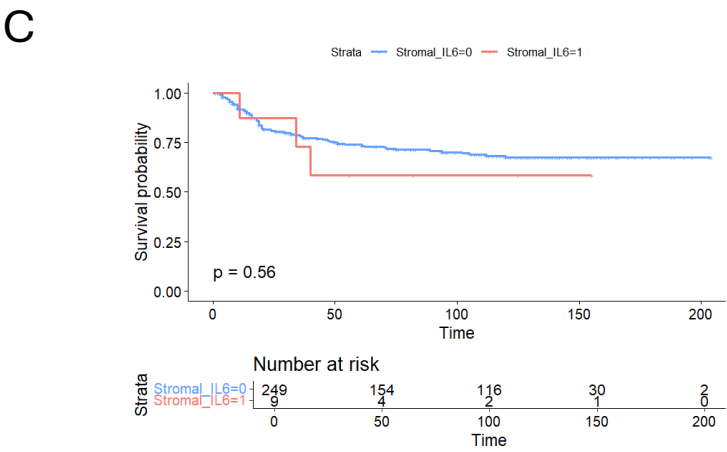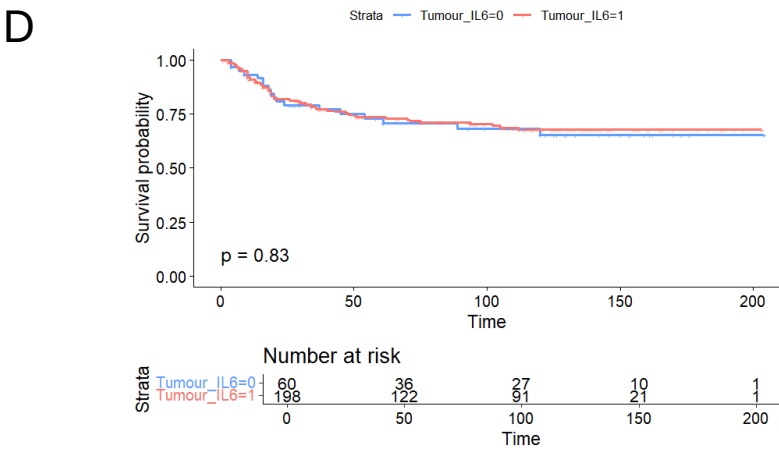

Supplement: Supplementary file 1 [file biomolecules-14-01629-s001.zip › Biomolecules Supplementary Figure 4.pdf]

A

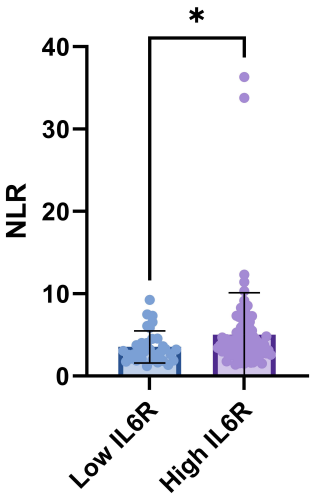

B

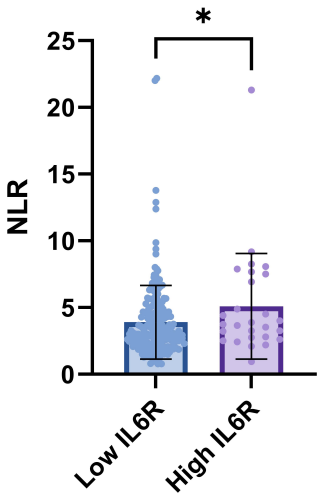

Supplement: Supplementary file 1 [file biomolecules-14-01629-s001.zip › Biomolecules_Supplementary Figure 2 (1).pdf]

Supplementary Figure 3

A

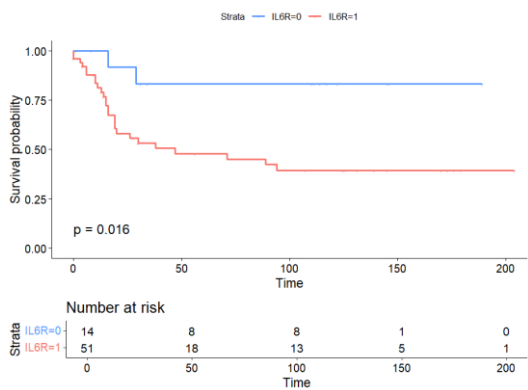

B

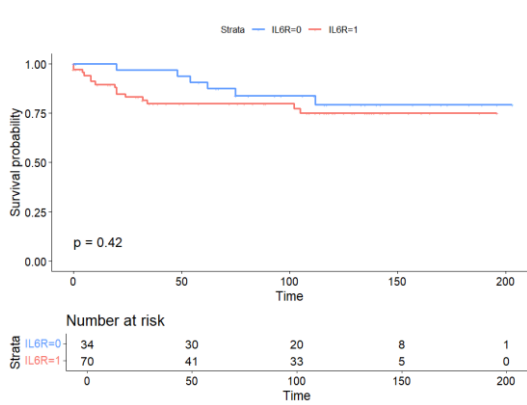

C

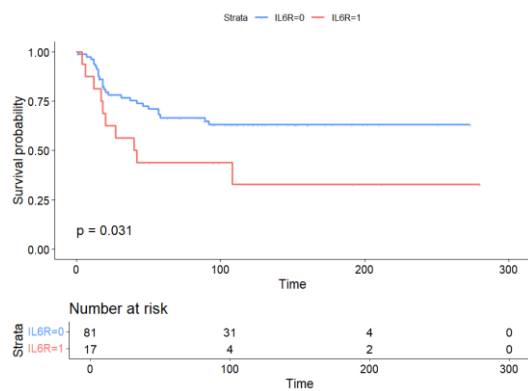

D

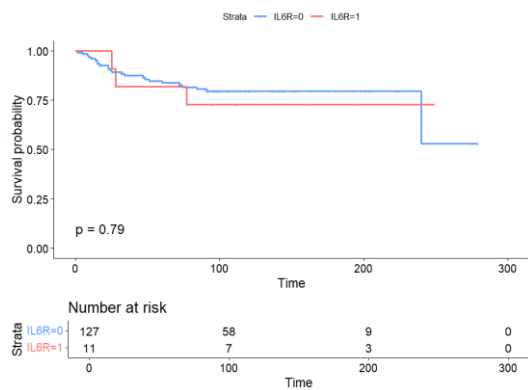

Supplement: Supplementary file 1 [file biomolecules-14-01629-s001.zip › Biomolecules_Supplementary Figure 3 (1).pdf]
